# Development of a new prognostic index PNPI for prognosis prediction of CKD patients with pneumonia at hospital admission

**DOI:** 10.3389/fmed.2023.1135586

**Published:** 2023-08-10

**Authors:** Xiao-Yu Cai, Jia-He Fan, Yi-Chun Cheng, Shu-Wang Ge, Gang Xu

**Affiliations:** Division of Internal Medicine, Department of Nephrology, Tongji Hospital, Tongji Medical College, Huazhong University of Science and Technology, Wuhan, China

**Keywords:** chronic kidney disease, pneumonia, clinical characteristics, risk factors, nomogram

## Abstract

**Background:**

The aim of this study was to investigate the relationship between pneumonia and chronic kidney disease (CKD), to elucidate potential risk factors, and to develop a new predictive model for the poor prognosis of pneumonia in CKD patients.

**Method:**

We conducted a retrospective observational study of CKD patients admitted to Tongji Hospital between June 2012 and June 2022. Demographic information, comorbidities or laboratory tests were collected. Applying univariate and multivariate logistic regression analyses, independent risk factors associated with a poor prognosis (i.e., respiratory failure, shock, combined other organ failure, and/or death during hospitalization) for pneumonia in CKD patients were discovered, with nomogram model subsequently developed. Predictive model was compared with other commonly used pneumonia severity scores.

**Result:**

Of 3,193 CKD patients with pneumonia, 1,013 (31.7%) met the primary endpoint during hospitalization. Risk factors predicting poor prognosis of pneumonia in CKD patients were selected on the result of multivariate logistic regression models, including chronic cardiac disease; CKD stage; elevated neutrophil to lymphocyte ratio (NLR) and D-dimer; decreased platelets, PTA, and chloride iron; and significant symptom presence and GGO presentation on CT. The nomogram model outperformed other pneumonia severity indices with AUC of 0.82 (95% CI: 0.80, 0.84) in training set and 0.83 (95% CI: 0.80, 0.86) in testing set. In addition, calibration curve and decision curve analysis (DCA) proved its efficiency and adaptability.

**Conclusion:**

We designed a clinical prediction model PNPI (pneumonia in nephropathy patients prognostic index) to assess the risk of poor prognosis in CKD patients with pneumonia, which may be generalized after more external validation.

## Introduction

Chronic kidney disease (CKD) also imposes a significant burden on health care systems. It is estimated that approximately 698 million people worldwide have CKD, with an estimated global prevalence of 9.1% in the adult population (1). CKD patients may be susceptible to pneumonia due to immunosuppressive therapy or the effects of uremic toxins. Previous researches have indicated that pneumonia was probably the most prevalent infection in these patients (2, 3). Pneumonia is three times more common in CKD patients than in those with normal renal function, respectively, and the length of hospitalization for pneumonia is four to six times greater than in those without CKD (2, 4, 5). A recent community-based research by James et al. (5) found that a decreased glomerular filtration rate was associated with an increased risk of pneumonia-related hospitalization and mortality. Despite these findings, the outcomes of patients with different courses of pneumonia (mild and severe) vary widely, and models for early and comprehensive assessment of prognostic stratification of pneumonia are lacking.

Given the notable occurrence of pneumonia in CKD population, risk stratification and death prediction may be key to prognosis improvement. Scoring systems combining multiple serum biomarkers and clinical parameters have been developed as a way to assess pneumonia and predict outcomes (6). For example, the Infectious Diseases Society of America/American Thoracic Society (IDSA/ATS) system, the Pneumonia Severity Score Index (PSI), score based on Confusion, Urea, Respiratory rate, Blood pressure, and age 65 (CURB 65); the Severe Community Acquired Pneumonia (SCAP) score, and the score based on Systolic blood pressure, Multilobar involvement, Albumin, Respiratory rate, Tachycardia, Confusion, Oxygenation, and PH (SMART-COP), which are now widely used to predict clinical outcomes in pneumonia (7–9). However, these systems have certain drawbacks, including the need for laboratory variables that are difficult to obtain on admission (10), performing poorly in predicting high-risk patients, and showing a low positive rate in predicting the recommended threshold for 30 days mortality (7). In addition, their accuracy and applicability in patients with CKD have decreased (11) and most are still not applicable in the case of pulmonary infections in CKD. Accurate prognosis of the patient’s severity enables clinicians to ascertain the suitable treatment site (outpatient vs. ward); furthermore, it facilitates the strength of care management, such as intravenous antibiotic medication and/or ICU admission (12). Therefore, novel, reliable and convenient predictive tools are urgently needed.

The aim of this study was to elucidate potential risk factors for the association between CKD and pneumonia outcomes, to develop a novel predictive model based on the clinical characteristics of patients at admission for severity risk assessment during hospitalization, and to compare the performance with other scoring systems currently in use as representative clinical assessment methods.

## Method

### Study design and participants

This retrospective observational research was conducted in an internal medicine inpatient unit of a large tertiary care hospital in Wuhan, China. The inclusion criteria for this study were patients with CKD hospitalized between June 2012 and June 2022 who (i) had an estimated glomerular filtration rate (eGFR) < 60 mL/min/1.73 m^2^ (applying the Chronic Kidney Disease Collaborative Epidemiological Equation) or a urinary albumin-creatinine ratio (ACR) > 30 mg/g within 48 h of admission (13) and (ii) aged over 18 years. Patients with acute kidney injury (AKI), comorbid chronic lung diseases (including chronic bronchitis, chronic obstructive pulmonary disease, bronchiectasis, interstitial pneumonia, follicular bronchitis, and a history of tuberculosis), pulmonary edema (caused by heart failure) on admission, HIV infection, viral hepatitis, *helicobacter pylori* infection, current cancer, or solid organ transplantation were excluded. These comorbidities were known to be associated with unique immune statuses that may increase the susceptibility of patients to pulmonary infections. We also excluded pregnant individuals and those with insufficient medical information. Due to Tongji Hospital’s policy, CKD patients with corona virus disease 2019 (COVID-19) were not included in this study. For controls with numerous hospitalizations over the research period, we only analyzed the initial admission. The need for informed patient consent was waived since the data set was comprised of de-identified secondary data used for research purposes. The Medical Ethics Committee at Tongji Hospital, Tongji Medical College, Huazhong University of Science & Technology authorized this study (Wuhan, Hubei, China; TJ-IRB20220503), which was done in line with the Helsinki Declaration.

### Pneumonia requiring hospitalization

Patients with CKD who had a discharge diagnostic of 480.0–487.7 (International Classification of Diseases [ICD]-9-CM values) or J10-J18 (ICD-10-CA values) were identified as pneumonia. These ICD diagnostic codes have more accuracy than medical records (14, 15) in detecting pneumonia (98% sensitivity and 97% specificity) and have been employed in several pneumonia investigations (16, 17). We studied only hospitalized patients with pulmonary infections based on the assumption that patients who present with pneumonia and are diagnosed with a pulmonary infection usually require hospital admission. A retrospective review of medical records was conducted. For patients who had multiple admissions for pulmonary infections during the study period, only the initial episode was evaluated.

### Primary endpoint

A combined primary endpoint (PE) was used, which was defined as the presence with one of the following: (i) respiratory failure, or the need for mechanical ventilation, (ii) shock, (iii) combination of other organ failure requiring ICU monitoring, and/or (iv) death. Pulmonary infection patients were further divided into PE and non-PE groups based on the outcome of the patients during their hospitalization.

### Data collection

The following clinical data were collected anonymously from the electronic medical record within 48 h of admission, including demographics, comorbidities, major symptoms (fever, cough, expectoration, chest pain or dyspnea) and vital signs on admission, relevant medications (use of glucocorticoids and other immunosuppressive agents before diagnosis of pneumonia), laboratory parameters (blood cell count, liver function indicators, renal function indicators, coagulation indicators, blood electrolytes, etc.), and medical imaging (chest computed tomography (CT) scan showing pulmonary infection manifestations such as ground glass changes (GGO), reticular shadow, consolidation, nodules in the lungs). Finally, we recorded outcomes, such as length of stay, and in-hospital mortality. Comorbidities were determined by prior ICD coding without time limit. For values that were truncated left or right, we used the value of the truncated point as a surrogate (e.g., 0.22 μg/mL for those with d-dimer levels <0.22 μg/mL).

Immunosuppressive agents were divided into two categories: glucocorticoids and immunomodulators. Immunomodulators are divided into four categories: calcineurinase inhibitors (cyclosporine and tacrolimus), antiproliferative agents (azathioprine, mycophenolate, cyclophosphamide, and methotrexate), monoclonal antibodies (rituximab and betriximab), and proprietary Chinese medicines (tripterygium glycosides, total Glucosides of paeony Capsules).

### Statistical analysis and model development

Age, gender, and comorbidities are regarded as risk factors for disease severity and mortality (18) and vary considerably between CKD patients with and without pneumonia. In particular, comorbidities include diabetes mellitus, hypertension, liver disease, cardiovascular disease and cerebrovascular disease. A propensity score matching (PSM) analytical model with a caliper of 0.05 was applied to equalize the differences in the aforementioned factors across groups, reduce the possibility of selection bias, and increase the evidence level of retrospective studies. Using the “nearest neighbor” matching model, statistically matched pairs of pneumonia patients were chosen according on age, sex, and comorbidities to reach a PSM ratio of 1:2.

Multiple imputation (MI) was employed to compute missing data if the proportion of missing values was less than 20%, whereas variables with missing rate more than 20% were removed. Continuous variables with a normal distribution were reported as mean ± standard deviation (SD) and examined using the Student’s *t*-test. Other continuous variables were reported as median and 25th or 75th interquartile range (IQR), and the Mann–Whitney U test was used to compare between-group variables. Using Chi-square or Fisher’s exact test, categorical variable differences were evaluated. Kaplan–Meier plots and logarithmic statistics were utilized to examine the predictive influence of CKD stage and related comorbidities on PE in CKD patients with pneumonia.

To determine risk factors related with the in-hospital primary endpoint, CKD patients with pneumonia were subsequently randomized into a training set (consisting of 70% of patients) for model building and a testing set (consisting of 30% of patients) for model validation. For further analysis and modeling, continuous variables were categorized, with thresholds defined by clinically relevant cutoffs or upper/lower limits of the normal range. In the training set, univariate logistic regression analysis was performed, where a significance level of *p* 0.05 was required for inclusion in multivariate analysis. Backward selection based on the Akaike Information Criterion (AIC) was implemented to exclude features from the prediction model. In the final model, only statistically significant factors (*p* < 0.05) were preserved, as estimated odds ratios (OR) and 95% confidence intervals (CI). A nomogram was then constructed using the final model equation. We evaluated the performance of predictive nomogram (19–21) by receiver operating characteristic (ROC) curve analysis, calibration curve, and decision curve analysis (DCA). ROC curve analyses of guideline-recommended scoring systems for assessing pneumonia severity, including CURB65, CRB65, PSI, CURXO, and SMART-COP, as well as ROC curve analyses of Halm, SCAP index, SIRS, qSOFA, and NEWS reported in other studies, were also performed to compare their potential power for PE prediction with the nomogram, which were presented as sensitivity, specificity, accuracy, area under the curve (AUC), and 95% CI. The sensitivity and specificity thresholds were obtained using Youden’s J statistic (22). AUCs of PNPI and other indices were compared using the method of DeLong et al. (23).

All two-tailed tests were statistically significant at *p* < 0.05. All statistical analyses were performed using RStudio software (version 1.1.423).

## Result

### Clinical characteristics of pneumonia patients associated with PE

**Figure 1:**
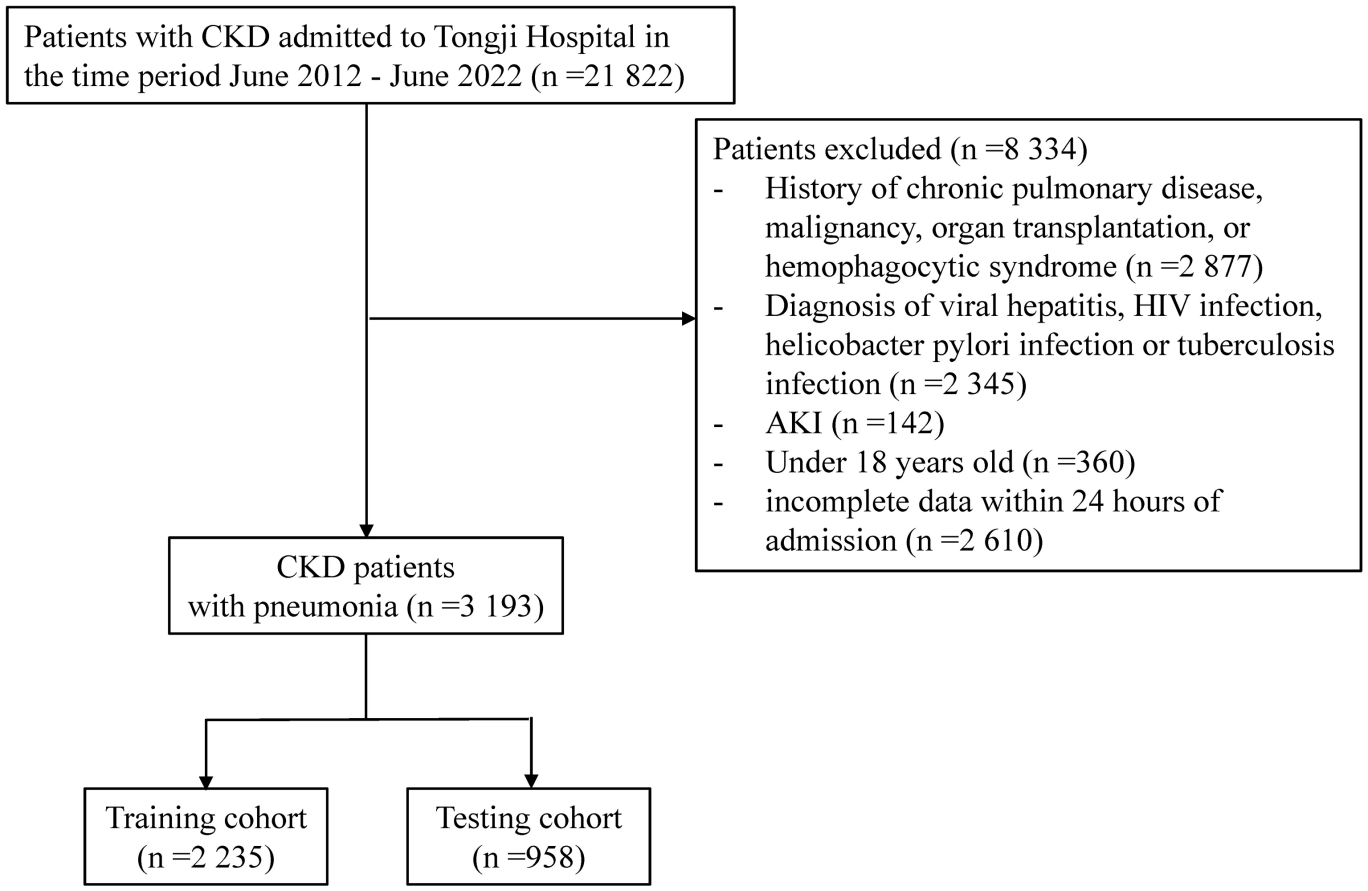
Enrollment of study population.

21,822 CKD patients were evaluated in the study. 8,334 patients were then eliminated based on exclusion criteria. Of the rest 13,488 patients with CKD, 3193 (23.7%) were diagnosed with pneumonia (Figure 1). The between-group differences between the pneumonia and non-pneumonia groups after PSM and the results of the multivariate logistic regression was shown in Supplementary Tables S1, S2. Table 1 compares the characteristics of the PE and non-PE groups at the time of pneumonia diagnosis. In general, most characteristics were significantly different between the PE and non-PE groups. Compared to the non-PE group, PE patients were older, more likely to smoke and consume alcohol, had a higher proportion of chronic comorbidities, and had more patients with >2 comorbidities at the same time. The comorbidities significantly associated with PE patients were chronic cardiac disease and hypertension. PE patients had more pneumonia-related symptoms and CT presentations on admission and worse associated vital signs. Comparing medication use, we found that PE patients were less likely to have previously used immunosuppressive drugs. In terms of laboratory tests, PE patients had significantly lower white blood cells, lymphocytes, hemoglobin, and platelets. The PE group had more severe abnormal liver and kidney function. Specifically, the PE group had significantly higher concentrations of total bilirubin, urea, and creatinine, while albumin levels and eGFR were lower. Correspondingly, inflammatory and coagulation markers were further increased in PE patients, indicating overactive inflammation and collapse of the coagulation system.

**Table 1:** The clinical characteristics among pneumonia patients with CKD.

	Non-PE (*n* = 2,180)	PE (*n* = 1,013)	*p* value
Demographic characteristics			
Sex: male (%)	1,183 (54.3)	551 (54.4)	0.977
Age, yrs. (mean (SD))	51.48 (16.99)	54.75 (17.21)	<0.001
Smoke (%)	607 (27.8)	337 (33.3)	0.002
Alcohol (%)	238 (10.9)	134 (13.2)	0.067
Comorbidities			
Hypertension (%)	1,153 (52.9)	668 (65.9)	<0.001
Diabetes mellitus (%)	488 (22.4)	258 (25.5)	0.061
Chronic cardiac diseases (%)	335 (15.4)	409 (40.4)	<0.001
Chronic cerebrovascular diseases (%)	120 (5.5)	92 (9.1)	<0.001
Chronic hepatic diseases (%)	138 (6.3)	81 (8.0)	0.097
Comorbidities (%)			<0.001
0	800 (36.7)	195 (19.2)	
1	728 (33.4)	326 (32.2)	
≥ 2	652 (29.9)	492 (48.6)	
Etiology of CKD			
Chronic nephritis syndrome^a^ (%)	989 (45.4)	375 (37.0)	<0.001
IgA nephropathy (%)	125 (5.7)	28 (2.8)	<0.001
Membranous nephropathy (%)	194 (8.9)	11 (1.1)	<0.001
Focal proliferative glomerulonephritis (%)	7 (0.3)	2 (0.2)	0.799
Tegumentary proliferative glomerulonephritis (%)	10 (0.5)	0 (0.0)	0.069
Focal segmental glomerulosclerosis (%)	7 (0.3)	0 (0.0)	0.162
Lupus nephritis (%)	219 (10.0)	78 (7.7)	0.040
Allergic purpura nephritis (%)	34 (1.6)	6 (0.6)	0.034
ANCA-associated nephritis (%)	107 (4.9)	53 (5.2)	0.762
Diabetic nephropathy (%)	142 (6.5)	79 (7.8)	0.209
Hypertensive nephropathy (%)	23 (1.1)	11 (1.1)	1.000
Hyperuric acid nephropathy (%)	6 (0.3)	4 (0.4)	0.824
Obstructive nephropathy (%)	9 (0.4)	8 (0.8)	0.271
Chronic tubulointerstitial disease (%)	6 (0.3)	1 (0.1)	0.558
Vital signs on admission			
Temperature, °C (%)			<0.001
< 37.0	1943 (89.1)	808 (79.8)	
≥ 37.0	237 (10.9)	205 (20.2)	
Heart rate, beat/min (%)			<0.001
< 100	1750 (80.3)	720 (71.1)	
≥ 100	430 (19.7)	293 (28.9)	
Blood pressure, mmHg (%)			<0.001
≥ 90/60	2,104 (96.5)	949 (93.7)	
< 90/60	76 (3.5)	64 (6.3)	
Primary symptoms			
Fever (%)	754 (34.6)	455 (44.9)	<0.001
Cough (%)	1,101 (50.5)	584 (57.7)	<0.001
Expectoration (%)	937 (43.0)	546 (53.9)	<0.001
Dyspnea (%)	539 (24.7)	458 (45.2)	<0.001
Chest pain (%)	191 (8.8)	138 (13.6)	<0.001
Radiological manifestation			
Ground glass opacity (%)	891 (40.9)	548 (54.1)	<0.001
Reticular shadow (%)	344 (15.8)	205 (20.2)	0.002
Pleural effusion (%)	838 (38.4)	658 (65.0)	<0.001
Immunosuppressive therapy			
Glucocorticoid (%)	1,367 (62.7)	594 (58.6)	0.031
Anti-proliferative agents^b^ (%)	551 (25.3)	141 (13.9)	<0.001
CNI^c^ (%)	234 (10.7)	42 (4.1)	<0.001
Biological agents (%)	87 (4.0)	21 (2.1)	0.007
Tripterygium glycosides (%)	125 (5.7)	24 (2.4)	<0.001
Hydroxychloroquine (%)	132 (6.1)	30 (3.0)	<0.001
Total Glucosides of paeony Capsules (%)	89 (4.1)	23 (2.3)	0.013
Laboratory parameters			
Neutrophil, 10^9^/L (median [IQR])	4.74 [3.30, 7.00]	5.12 [3.34, 7.96]	0.001
Lymphocyte, 10^9^/L (median [IQR])	1.16 [0.77, 1.66]	0.77 [0.48, 1.12]	<0.001
NLR (%)			<0.001
< 7	1,633 (74.9)	559 (55.2)	
7–19	448 (20.6)	287 (28.3)	
> 19	99 (4.5)	167 (16.5)	
Monocyte, 10^9^/L (median [IQR])	0.51 [0.36, 0.71]	0.53 [0.34, 0.78]	0.162
White blood cell, 10^9^/L (median [IQR])	6.43 [4.91, 8.51]	5.92 [4.37, 7.85]	<0.001
Platelet, 10^9^/L (%)			<0.001
≥ 100	1993 (91.4)	758 (74.8)	
< 100	187 (8.6)	255 (25.2)	
Hemoglobin, g/L (%)			<0.001
< 80	478 (21.9)	525 (51.8)	
80–100	596 (27.3)	300 (29.6)	
> 100	1,106 (50.7)	188 (18.6)	
LDH, U/L (%)			<0.001
< 215	806 (37.0)	199 (19.6)	
≥ 215	1,374 (63.0)	814 (80.4)	
BUN, mmol/L (median [IQR])	12.00 [6.60, 20.72]	21.40 [13.90, 30.02]	<0.001
Uric acid, umol/L (mean (SD))	414.61 (145.94)	444.43 (158.56)	<0.001
Creatinine, umol/L (median [IQR])	186.00 [88.75, 499.00]	534.00 [284.00, 794.00]	<0.001
CKD stage (%)			<0.001
1	348 (16.0)	29 (2.9)	
2	328 (15.0)	36 (3.6)	
3	377 (17.3)	87 (8.6)	
4	291 (13.3)	112 (11.1)	
5	836 (38.3)	749 (73.9)	
Total bilirubin, umol/L (median [IQR])	4.90 [3.50, 7.20]	5.60 [3.80, 8.50]	<0.001
Globulin, g/L (median [IQR])	29.70 [25.40, 36.00]	29.10 [24.90, 34.80]	0.011
Albumin, g/L (mean (SD))	34.21 (12.83)	31.61 (10.49)	<0.001
Calcium iron, mmol/L (mean (SD))	2.06 (0.22)	1.97 (0.24)	<0.001
Chlorine iron, mmol/L (%)			<0.001
< 97	559 (25.6)	464 (45.8)	
97–102	761 (34.9)	366 (36.1)	
> 102	860 (39.4)	183 (18.1)	
Sodium iron, mmol/L (median [IQR])	137.50 [133.70, 140.00]	135.70 [131.60, 138.40]	<0.001
Potassium iron, mmol/L (mean (SD))	4.51 (0.74)	4.90 (0.90)	<0.001
ACR, ug/mg (median [IQR])	1691.50 [447.48, 3789.00]	2074.60 [696.10, 4301.00]	<0.001
D-dimer, mg/L (%)			<0.001
< 1	1,016 (46.6)	157 (15.5)	
≥ 1	1,164 (53.4)	856 (84.5)	
INR (median [IQR])	1.02 [0.95, 1.10]	1.13 [1.04, 1.25]	<0.001
PT, s (median [IQR])	13.30 [12.70, 14.10]	14.40 [13.50, 15.60]	<0.001
PTA, % (%)			<0.001
75–125	1832 (84.0)	660 (65.2)	
< 75	201 (9.2)	340 (33.6)	
> 125	147 (6.7)	13 (1.3)	
TT, s (median [IQR])	17.10 [16.20, 18.40]	17.20 [16.30, 18.70]	0.018
APTT, s (median [IQR])	38.20 [34.68, 42.70]	42.20 [37.60, 48.70]	<0.001
Fibrinogen, g/L (mean (SD))	4.94 (1.77)	5.06 (1.81)	0.061
Urine ph (mean (SD))	6.48 (0.80)	6.54 (0.88)	0.057
Urine WBC, /ul (median [IQR])	14.25 [6.00, 41.00]	23.20 [9.00, 58.90]	<0.001
Urine RBC, /ul (median [IQR])	29.85 [10.60, 102.00]	39.90 [12.60, 154.00]	<0.001
Clinical outcomes			
LOS, d (median [IQR])	10.00 [7.00, 14.00]	12.00 [8.00, 19.00]	<0.001
Total costs, yuan (median [IQR])	13917.20 [9067.44, 24335.66]	36200.11 [20260.63, 72557.19]	<0.001
Death within 14 days (%)	0 (0.0)	262 (25.9)	<0.001
Death within 30 days (%)	0 (0.0)	415 (41.0)	<0.001
Hospital mortality (%)	0 (0.0)	460 (45.4)	<0.001

Data were presented by numbers (%) or median (IQR) or mean (SD). PE, primary endpoint; ANCA, anti-neutrophil cytoplasmic antibodies; CNI, calcineurin inhibitor; NLR, neutrophil to lymphocyte ratio; LDH, lactate dehydrogenase; BUN, blood urea nitrogen; eGFR, estimated glomerular filtration rate; ACR, urine albumin creatinine ratio; INR, international normalized ratio; PT, prothrombin time; PTA, prothrombin time activity; TT, thrombin time; APTT, activated partial thromboplastin time; WBC, white blood cell; RBC, red blood cell; LOS, length of stay; IQR, interquartile range; SD, standard Deviation.

a Chronic nephritis syndrome represented patients without pathological diagnosis.

b Anti-proliferative agents include mycophenolate mofetil, leflunomide, cyclophosphamide, methotrexate, and azathioprine.

c Calcineurin inhibitor include cyclosporine and tacrolimus.

**Figure 2:**
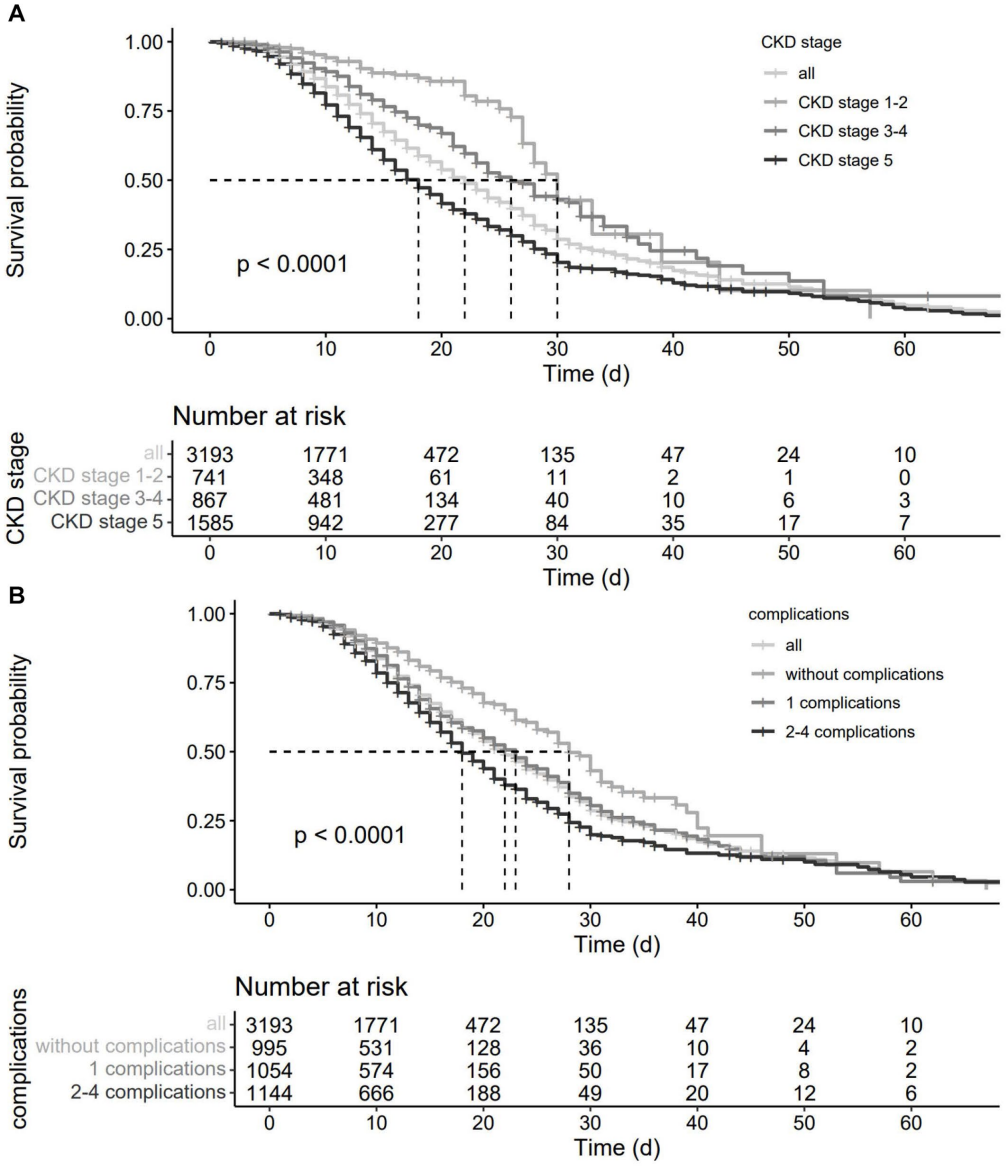
Survival curves of CKD patients with pneumonia.(**A**), grouped by CKD stage; (**B**), grouped by clinical complications.

Kaplan–Meier curves showed (Figure 2) that CKD staging status and associated complications prior to hospital admission had a significant negative effect on the prognosis of pneumonia patients (*p* < 0.001).

### Development of a predictive model in CKD patients with pneumonia

**Figure 3:**
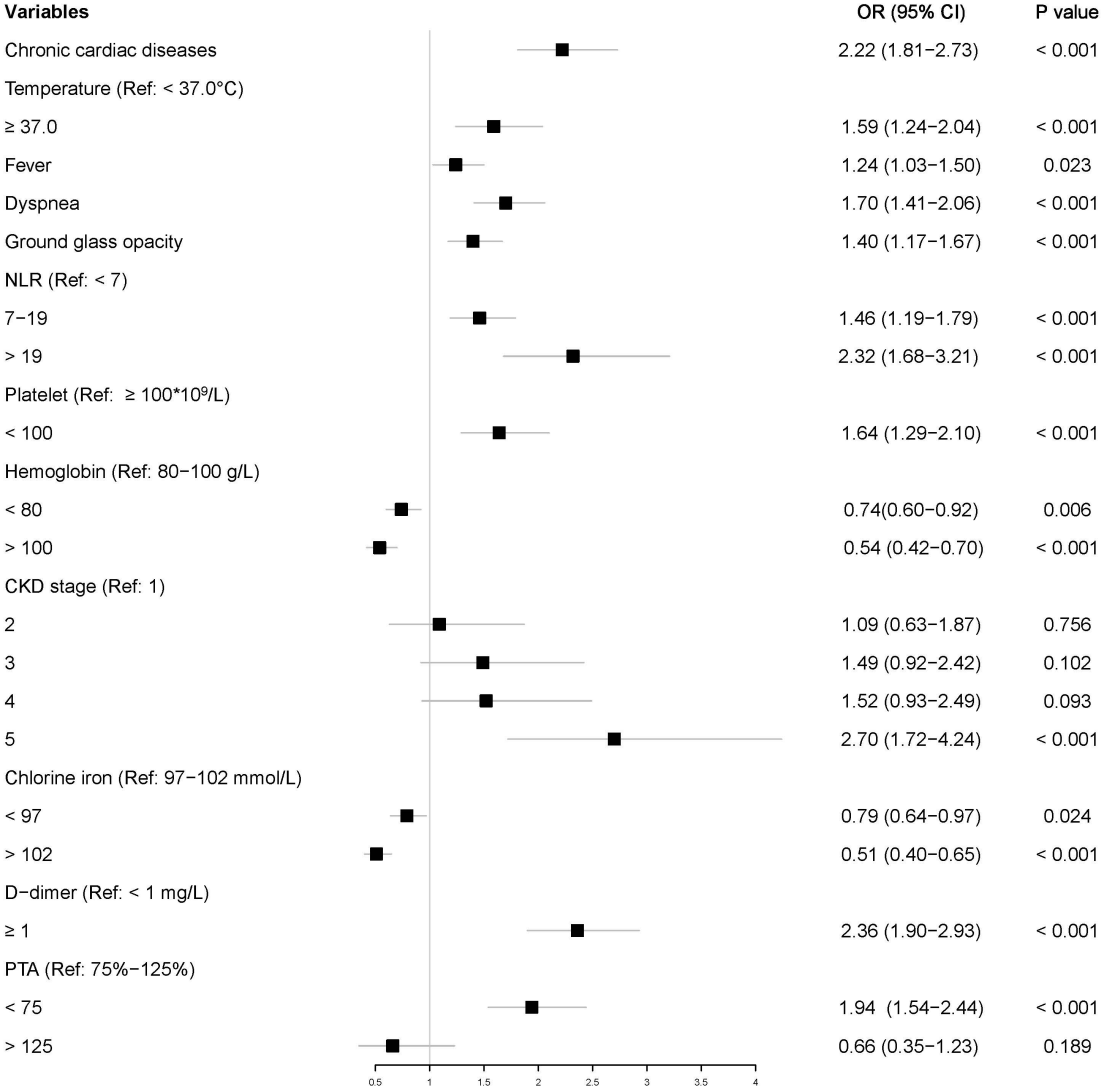
Independent risk factors for primary endpoint in the training set.

Of the 3,193 patients diagnosed with pneumonia, 2,235 were randomly assigned to the training set and 958 to the testing set. In multivariate analysis, variables having *p* values less than 0.05 in univariate logistic regression analyses were considered. Multivariate analysis identified 12 characteristics as independent risk factors: chronic cardiac disease; elevated neutrophil to lymphocyte ratio (NLR) and d-dimer; decreased platelets, hemoglobin, chloride, PTA, and eGFR; and elevated temperature, present symptoms, and CT manifestations. Figure 3 showed the ORs with 95% CIs.

**Figure 4:**
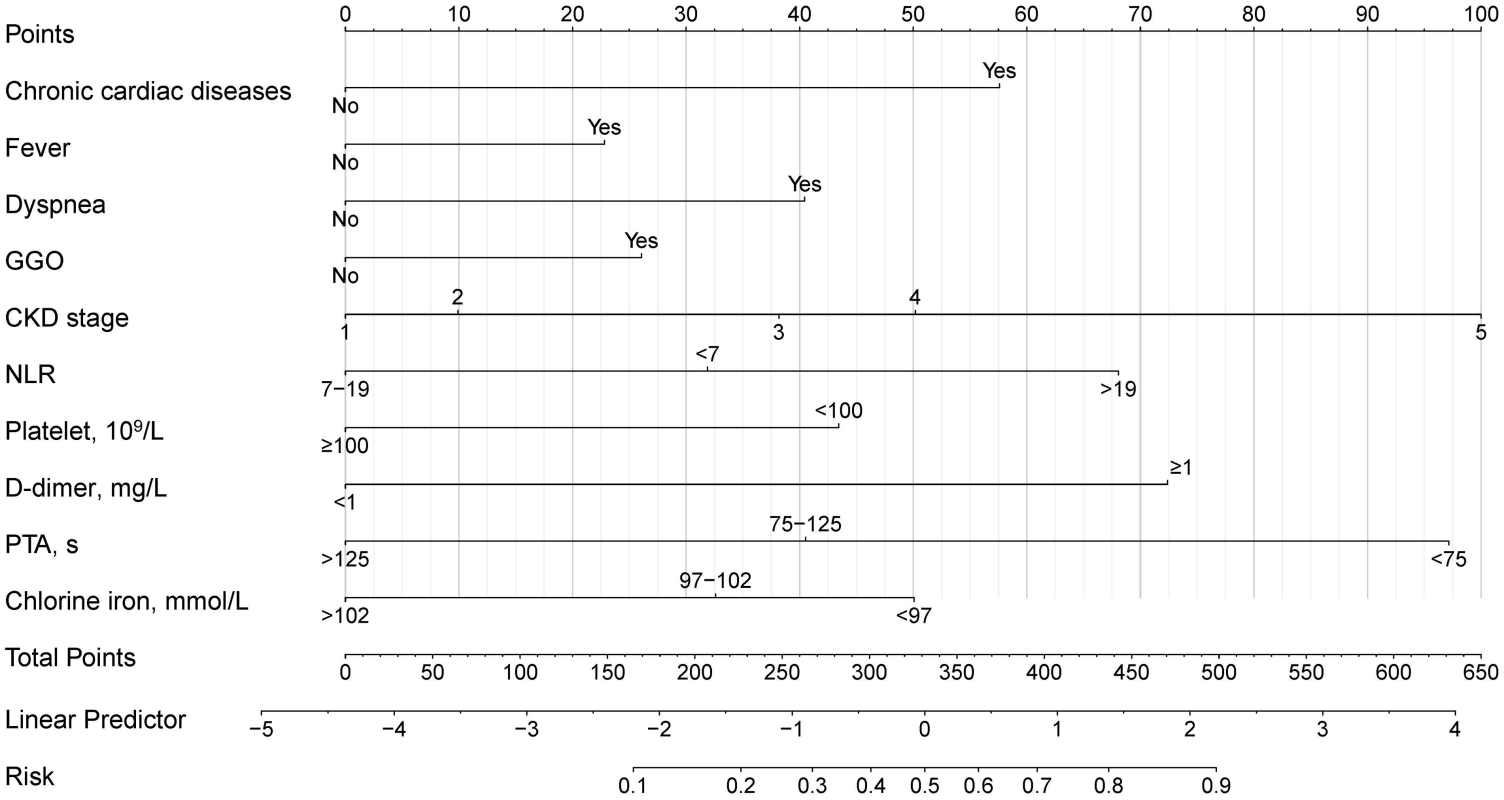
The nomogram for primary endpoint in CKD patients with pneumonia.

To develop a simple and useful clinical prediction tool, ten of these factors were selected for inclusion in the nomogram (Figure 4). For each patient, the summed score of each indicator corresponded to the risk at the bottom of the nomogram, which predicted the probability of PE in CKD patients with pneumonia. The prognostic score was named PNPI (pneumonia in nephropathy patients prognostic index).

### Evaluation and validation of the PNPI

**Figure 5:**
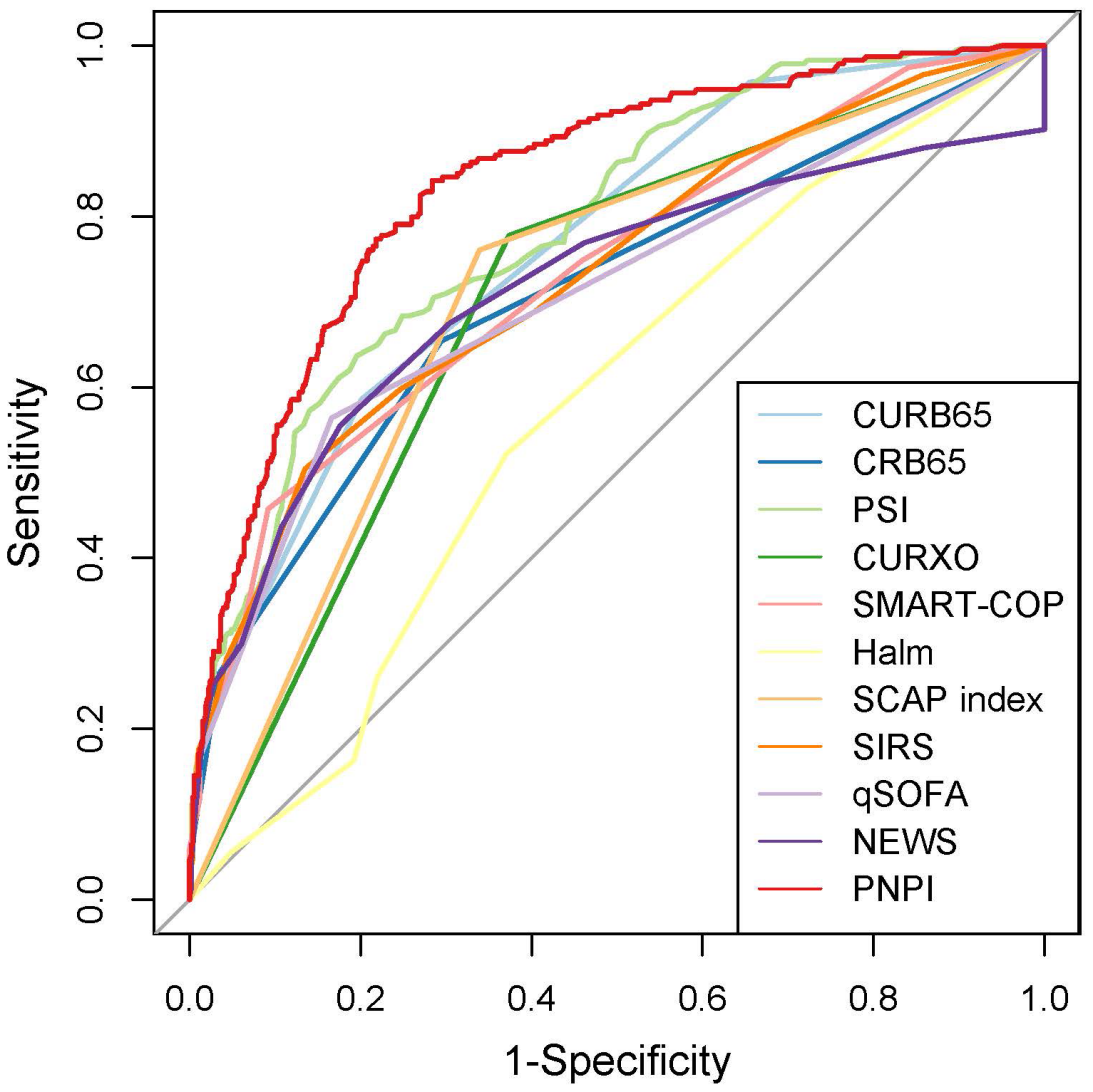
Receiver operating characteristics for severity scores at enrollment regarding PE prediction in testing set.

**Figure 6:**
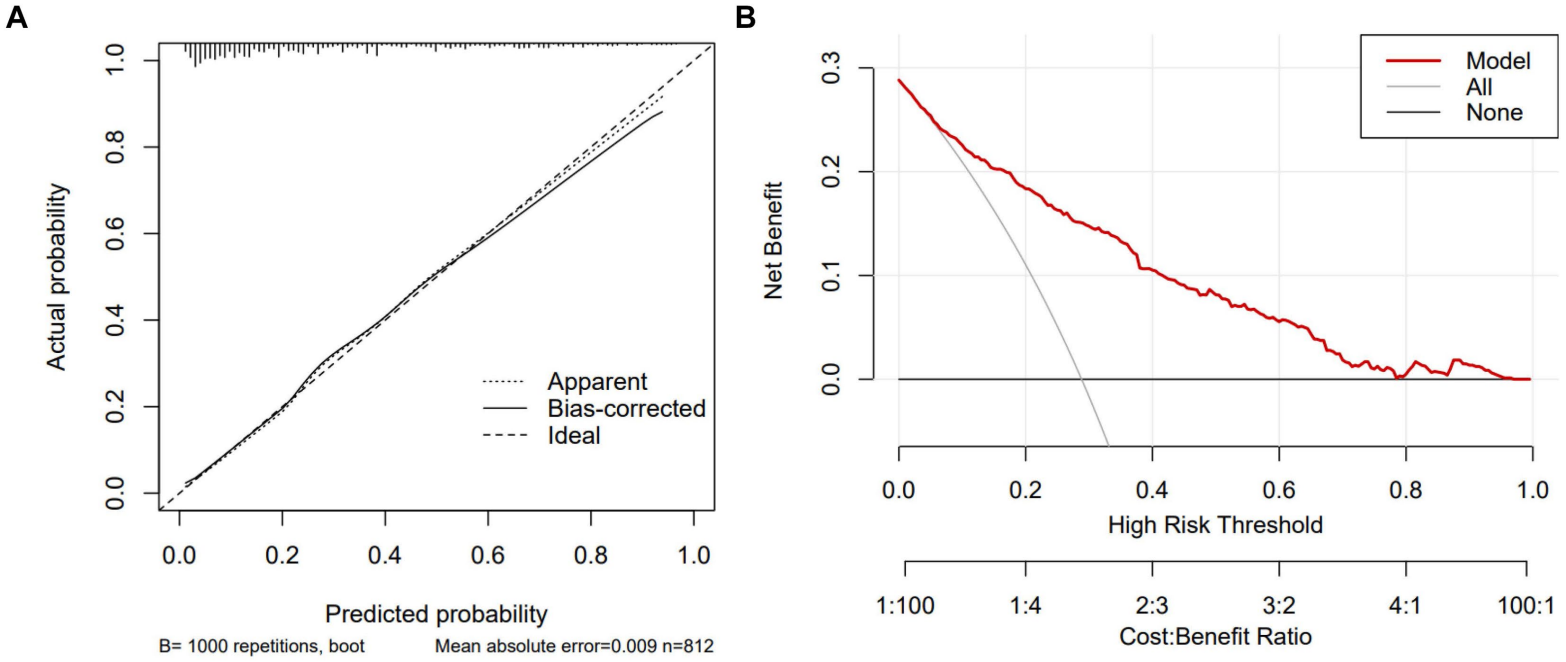
Calibration curve and DCA of PNPI in testing set. DCA, decision curve analysis. **(A)** Calibration curve. **(B)** DCA.

In the test group, 305 patients (31.8%) developed PE with AUC of 0.83 (95% CI: 0.80, 0.86), indicating the good predictive value of PNPI. ROC curve was shown in Figure 5, where a score of 270.0 was used as the cut-off value for the high-risk stratification of critical illness pneumonia. The model showed a sensitivity of 0.78 and specificity of 0.74. Supplementary Figure S4 shows the performance of the model for predicting the severity of PE in pneumonia patients in the study cohort, divided into 3 risk classes. The PE rate was 2.3% for the low-risk group (<130 points), 19.5% for the moderate-risk group (130–270 points), and 78.2% for the high-risk group (>270 points) (Supplementary Figure S1). The calibration curves did not depart significantly from the reference line, indicating an excellent correlation between the anticipated and observed values of the PNPI (Figure 6A). The DCA demonstrated that the PNPI had a positive net benefit over a broad spectrum of threshold probabilities (Figure 6B).

Using the bootstrap method, the AUC for the training set was 0.82 (95% CI: 0.80, 0.84). ROC curve was shown in Supplementary Figure S2 with a sensitivity of 0.78 and a specificity of 0.72. The calibration curve and DCA were shown in Supplementary Figure S3.

In addition, to assess the operational potential of PNPI for PE prediction, 10 given scores were compared. The sensitivity, specificity, accuracy, AUC and its 95% confidence interval of the other pneumonia severity indices in pneumonia patients, as well as the results of the paired DeLong test between the ROC curves of PNPI and the other scores are shown in Table 2. The results indicated that the diagnostic value of PNPI is significantly better than the other scores, expressed as the maximum AUC.

**Table 2:** Diagnostic power of scores regarding the primary endpoint.

Dataset	Indicators	CURB65	CRB65	PSI	CURXO	SMART-COP	Halm	SCAP index	SIRS	qSOFA	NEWS	PNPI
Training set	Sensitivity	0.54	0.60	0.70	0.78	0.45	0.89	0.75	0.57	0.51	0.64	0.78
	Specificity	0.81	0.69	0.72	0.65	0.93	0.18	0.69	0.79	0.82	0.71	0.72
	Accuracy	0.73	0.66	0.72	0.69	0.79	0.39	0.71	0.72	0.73	0.69	0.74
	AUC	0.74 (0.71–0.76)	0.67 (0.64–0.69)	0.79(0.77–0.81)	0.72 (0.70–0.74)	0.75 (0.73–0.78)	0.45 (0.43–0.48)	0.72 (0.70–0.74)	0.74 (0.72–0.77)	0.67 (0.65–0.70)	0.71 (0.68–0.74)	0.82 (0.80–0.84)
	*p* value^a^	<0.001	<0.001	<0.001	<0.001	<0.001	<0.001	<0.001	<0.001	<0.001	<0.001	–
Testing set	Sensitivity	0.59	0.65	0.64	0.78	0.46	0.48	0.76	0.50	0.56	0.56	0.78
	Specificity	0.80	0.71	0.80	0.63	0.91	0.37	0.66	0.87	0.83	0.82	0.74
	Accuracy	0.74	0.69	0.76	0.67	0.78	0.40	0.69	0.76	0.76	0.75	0.75
	AUC	0.76 (0.73–0.80)	0.71 (0.67–0.75)	0.79 (0.75–0.82)	0.70 (0.67–0.74)	0.73 (0.69–0.77)	0.58 (0.53–0.62)	0.71 (0.68–0.74)	0.73 (0.69–0.77)	0.71 (0.67–0.75)	0.71 (0.67–0.76)	0.83 (0.80–0.86)
	*p* value^a^	<0.001	<0.001	0.003	<0.001	<0.001	<0.001	<0.001	<0.001	<0.001	<0.001	–

a The paired DeLong test for AUC of PNPI and other scores.

## Discussion

In our study population, the burden of pneumonia in CKD patients was heavy. After controlling for common confounding variables, CKD stage and comorbidities had a substantial negative effect on the prognosis of pneumonia patients. We developed and validated the predictive model PNPI for in-hospital primary endpoint based on 10 independent risk factors (i.e., chronic cardiac disease, CKD stage, fever, dyspnea, GGO, NLR, platelets, D-dimer, PTA, and serum chloride ions). In testing set, PNPI was validated to be superior to other commonly used predictive indices of pneumonia severity such as PSI. We believe that PNPI is useful for early risk assessment and triage of pneumonia in CKD patients, and for those patients with relatively high probability of adverse prognosis, clinicians can take more interventions at an earlier stage.

Our study uncovered a number of independent risk factors that have a significant association in the adverse outcome of CKD patients with pneumonia. In CKD patients with pneumonia, the predictive values of comorbidities such as chronic cardiac disease and typical symptoms and CT presentation on admission are consistent with prior studies reported in CAP patients with other diseases and in general CAP patients (24–26). In the context of previous cardiac disease, pneumonia can lead to rapid dysregulation of vital signs and function and also to acute vascular complications (27), which in part accelerates death in CKD patients. Therefore, chronic cardiac disease could be a strong predictor of poor outcome in pneumonia patients. In terms of laboratory tests, elevated NLR represented increased neutrophils and decreased lymphocytes, reflecting not only a systemic inflammatory storm *in vivo*, but also an impaired immune response, which could potentially surmount the disadvantage that absolute values might be impacted by conditions like dehydration. On one hand, different studies have shown that NLR was significantly related to inpatient and ICU death in pneumonia patients (28, 29). Yukai Huang et al. also reported that NLR was significantly associated with PSI, indicating disease severity in CAP (30). On the other hand, CKD was featured by innate and adaptive immune system disorders. Evidence suggested that immune dysfunction worsen with a decrease in GFR (31). Thus, elevated NLR at admission in patients with CKD might be a sign of insufficient immune response or severity of the disease, which was closely related to the prognosis of pneumonia. As another common indicator of inflammation, D-dimer is a small fragment protein byproduct of catabolic clots, indicating a hypercoagulable state resulting from a severe inflammatory response (32). The association of elevated D-dimer with adverse outcomes in pneumonia has been widely found and demonstrated on admission or post-admission sequential evaluation (33). Furthermore, in consistency with previous studies on CAP (34, 35), our results indicated that reduced platelets and PTA were independently associated with poor prognosis of pneumonia patients. Abnormalities in coagulation indicators such as D-dimers, platelets, and PTA indicate an increased risk of disseminated intravascular coagulation, which is one of the complications frequently diagnosed in late pneumonia and leads to poor prognosis. In addition, our study revealed that blood chloride level was an important prognostic indicator in pneumonia patients, which is not common in other studies on patients with pneumonia. We speculate that this might be due to the unique pathological metabolic state of CKD patients and needs to be further confirmed in clinical researches.

The application of nomogram to evaluate the probability of adverse prognosis in CKD patients with pneumonia is a novel concept based on the aforesaid risk factors. Our intuitive and user-friendly prediction model enables early and reliable identification of individuals at high risk prior to clinical decompensation. Compared to existing pneumonia prediction index, PNPI has a higher AUC and better net DCA benefit in the testing set. We suggest that the model can be programmed into hospital medical record systems and used as an initial screening and triage tool on admission to determine the likelihood of developing critical illness, providing an essential guidance for clinical decision making and optimum resource allocation. Also, the application of this predictive model is of great importance in primary care hospitals where critical care is not well developed. Once a patient is admitted, doctors may quickly evaluate the chance of a poor prognosis, which is important when choosing whether to transfer the patient to a higher level hospital. In addition, the model can be used for patient selection or stratification in clinical trials to homogenize patient populations.

On the basis of these findings, efforts should be made to investigate more rigorous and customized surveillance strategies and work to improve therapeutic and preventive approaches of pneumonia in CKD patients. However, some concerns deserve more investigation. Knowledge gaps still existed in the current research regarding the biological mechanisms underlying the association between urine protein or serum creatinine or CKD stage and pneumonia outcome, as well as the clinical features of pneumonia in different types of CKD. Furthermore, there is a lack of clear recommendations regarding the optimal cut-off values for these indicators of the model. Further research is required to update or recalibrate our model in order to more correctly evaluate the severity risk.

The relationship between CKD and pneumonia may be mediated by the following factors. Evidence has found that patients with CKD have direct or indirect pulmonary insufficiency due to circulating uremic toxins, volume overload, anemia, immunosuppression, extraosseous calcification, or malnutrition (36, 37), which may be associated with severe pneumonia. Subsequently, research has reported that impaired monocyte/macrophage (Mphi) stress response in CKD patients may lead to the observed immune dysfunction, which increases susceptibility to infection (38). We could speculate, that impaired immune competenceand dysfunctional pulmonary host defenses appear to be crucial in the poor prognosis of pneumonia. For example, fibroblast growth factor 23 (FGF23, a bone-derived endocrine hormone) can inhibit neutrophil adhesion and transendothelial migration and may also contribute to an increased risk of infectiousness by compromising the innate immune capabilities of monocytes (39). The Hemodialysis (HEMO) research found that greater FGF23 levels were significantly related with infection death (40). Furthermore, obesity and other lifestyle variables, which significantly impact immunological function and host defense systems, may be implicated (41). In addition, the actual correlation between CKD and pneumonia prognosis requires further validation after elimination of other possible confounding variables, including previous pneumococcal vaccine use, duration of symptoms before hospitalization, and CKD treatment.

Our study has several limitations. First, this was a single-center retrospective study with limited sample size, thus inherent bias is inevitable. Undiagnosed pneumonia may exist, which could skew or misclassify our findings. Although patients were split into training and testing groups, the nomogram was derived at the specific time and place where the data were collected. Our prediction model does not have additional data from external validation studies, and thus the robustness of the results cannot be formally determined. Second, the results may be influenced by unadjusted confounding variables like prior pneumococcal vaccination and patient self-treatment prior to hospital admission. Third, dynamic lab and clinical data was absent due to the scarcity of pertinent information and were not able to follow up after hospital discharge.

In conclusion, CKD patients with pneumonia have prominent clinical features and poor outcome. A subgroup of individuals with a higher stage of CKD and more comorbidities have a worse prognosis. We developed a clinically applicable model PNPI for assessing the severity of pneumonia in CKD patients. Our model has good predictive performance for patients with a high probability of death at admission and may be generalized after additional external validation to provide a basis for individualized interventions.

## Data availability statement

The raw data supporting the conclusions of this article will be made available by the authors, without undue reservation.

## Ethics statement

The study was approved by the Institutional Ethics Committee of Tongji Hospital.

## Author contributions

X-YC and J-HF designed the study, conducted the statistical analyses, and wrote the paper. Y-CC contributed to data interpretation and the critical review of the manuscript. S-WG and GX designed the study, supervised the study process, and contributed to data interpretation and the critical review of the manuscript. All authors contributed to the article and approved the submitted version.

## Funding

This work was financially supported by Project supported by the National Natural Science Foundation of China (Grants Nos. 82170702, 82230021, 81602104, 82100730, 82200771), Major Research plan of the National Natural Science Foundation of China (Grant No. 91742204), National Key R&D Program of China (Grant No. 2021YFC2500200).

## Conflict of interest

The authors declare that the research was conducted in the absence of any commercial or financial relationships that could be construed as a potential conflict of interest.

## Publisher’s note

All claims expressed in this article are solely those of the authors and do not necessarily represent those of their affiliated organizations, or those of the publisher, the editors and the reviewers. Any product that may be evaluated in this article, or claim that may be made by its manufacturer, is not guaranteed or endorsed by the publisher.
