# Supplementary material for: Development of a new prognostic index PNPI for prognosis prediction of CKD patients with pneumonia at hospital admission
Source: Front Med (Lausanne). 2023 Aug 10;10:1135586. doi: 10.3389/fmed.2023.1135586 (PMC10448187; doi:10.3389/fmed.2023.1135586)

Supplementary Table 1 Baseline clinical characteristics of CKD patients with or without pneumonia.

|  | Overall (n=8039) | CKD without pneumonia (n=5011) | CKD with pneumonia (n=2706) | p value |
| --- | --- | --- | --- | --- |
| Demographic characteristics |  |  |  |  |
| Sex: male (%) | 4155 (53.8) | 2704 (54.0) | 1451 (53.6) | 0.794 |
| Age, yrs (mean (SD)) | 50.41 (16.38) | 50.18 (16.29) | 50.84 (16.54) | 0.094 |
| Smoke (%) | 2213 (28.7) | 1446 (28.9) | 767 (28.3) | 0.654 |
| Alcohol (%) | 984 (12.8) | 682 (13.6) | 302 (11.2) | 0.002 |
| Comorbidities |  |  |  |  |
| Hypertension (%) | 4063 (52.6) | 2616 (52.2) | 1447 (53.5) | 0.298 |
| Diabetes mellitus (%) | 1590 (20.6) | 1013 (20.2) | 577 (21.3) | 0.263 |
| Chronic cardiac diseases (%) | 587 (7.6) | 284 (5.7) | 303 (11.2) | <0.001 |
| Chronic cerebrovascular diseases (%) | 359 (4.7) | 214 (4.3) | 145 (5.4) | 0.035 |
| Chronic hepatic diseases (%) | 466 (6.0) | 289 (5.8) | 177 (6.5) | 0.190 |
| Etiology of CKD | |  |  |  |
| Chronic nephritis syndrome^‡^ (%) | 2505 (32.5) | 1496 (29.9) | 1009 (37.3) | <0.001 |
| IgA nephropathy (%) | 583 (7.6) | 443 (8.8) | 140 (5.2) | <0.001 |
| Membranous nephropathy (%) | 401 (5.2) | 204 (4.1) | 197 (7.3) | <0.001 |
| Focal proliferative glomerulonephritis (%) | 59 (0.8) | 51 (1.0) | 8 (0.3) | 0.001 |
| Tegumentary proliferative glomerulonephritis (%) | 56 (0.7) | 46 (0.9) | 10 (0.4) | 0.01 |
| Focal segmental glomerulosclerosis (%) | 21 (0.3) | 14 (0.3) | 7 (0.3) | 1.000 |
| Familial recurrent hematuria syndrome (%) | 8 (0.1) | 8 (0.2) | 0 (0.0) | 0.087 |
| Lupus nephritis (%) | 414 (5.4) | 136 (2.7) | 278 (10.3) | <0.001 |
| Allergic purpura nephritis (%) | 111 (1.4) | 72 (1.4) | 39 (1.4) | 1.000 |
| ANCA-associated nephritis (%) | 179 (2.3) | 43 (0.9) | 136 (5.0) | <0.001 |
| Diabetic nephropathy (%) | 527 (6.8) | 361 (7.2) | 166 (6.1) | 0.084 |
| Hypertensive nephropathy (%) | 106 (1.4) | 83 (1.7) | 23 (0.8) | 0.005 |
| Hyperuric acid nephropathy (%) | 55 (0.7) | 45 (0.9) | 10 (0.4) | 0.013 |
| Obstructive nephropathy (%) | 35 (0.5) | 21 (0.4) | 14 (0.5) | 0.663 |
| Chronic tubulointerstitial disease (%) | 41 (0.5) | 36 (0.7) | 5 (0.2) | 0.004 |
| Vital signs on admission |  |  |  |  |
| Temperature, °C (median [IQR]) | 36.50 [36.30, 36.70] | 36.50 [36.30, 36.70] | 36.50 [36.30, 36.80] | <0.001 |
| Respiratory rate, breath/min (median [IQR]) | 20.00 [20.00, 20.00] | 20.00 [20.00, 20.00] | 20.00 [20.00, 20.00] | <0.001 |
| Heart rate, beat/min (mean (SD)) | 84.38 (14.26) | 82.45 (12.45) | 87.96 (16.52) | <0.001 |
| Systolic blood pressure, mmHg (mean (SD)) | 142.08 (26.39) | 143.36 (26.51) | 139.71 (26.00) | <0.001 |
| Diastolic blood pressure, mmHg (mean (SD)) | 86.64 (16.13) | 87.34 (16.24) | 85.34 (15.85) | <0.001 |
| Primary symptoms |  |  |  |  |
| Fever (%) | 1117 (14.5) | 89 (1.8) | 1028 (38.0) | <0.001 |
| Cough (%) | 1463 (19.0) | 60 (1.2) | 1403 (51.8) | <0.001 |
| Expectoration (%) | 1264 (16.4) | 19 (0.4) | 1245 (46.0) | <0.001 |
| Dyspnea (%) | 847 (11.0) | 84 (1.7) | 763 (28.2) | <0.001 |
| Chest pain (%) | 272 (3.5) | 22 (0.4) | 250 (9.2) | <0.001 |
| Radiological manifestation |  |  |  |  |
| Ground glass opacity (%) | 1327 (17.2) | 124 (2.5) | 1203 (44.5) | <0.001 |
| Reticular shadow (%) | 513 (6.6) | 45 (0.9) | 468 (17.3) | <0.001 |
| Pleural effusion (%) | 1566 (20.3) | 348 (6.9) | 1218 (45.0) | <0.001 |
| Immunosuppressive therapy |  |  |  |  |
| Glucocorticoid (%) | 3331 (43.2) | 1620 (32.3) | 1711 (63.2) | <0.001 |
| Anti-proliferative agents^*^ (%) | 1209 (15.7) | 561 (11.2) | 648 (23.9) | <0.001 |
| CNI^†^ (%) | 434 (5.6) | 175 (3.5) | 259 (9.6) | <0.001 |
| Biological agents (%) | 131 (1.7) | 30 (0.6) | 101 (3.7) | <0.001 |
| Tripterygium glycosides (%) | 319 (4.1) | 180 (3.6) | 139 (5.1) | 0.001 |
| Hydroxychloroquine (%) | 217 (2.8) | 67 (1.3) | 150 (5.5) | <0.001 |
| Total Glucosides of paeony Capsules (%) | 169 (2.2) | 64 (1.3) | 105 (3.9) | <0.001 |
| Laboratory parameters |  |  |  |  |
| Neutrophil, 10^9^/L (median [IQR]) | 4.40 [3.26, 6.08] | 4.24 [3.25, 5.60] | 4.78 [3.28, 7.22] | <0.001 |
| Lymphocyte, 10^9^/L (median [IQR]) | 1.30 [0.88, 1.79] | 1.42 [1.02, 1.89] | 1.05 [0.67, 1.58] | <0.001 |
| NLR (median [IQR]) | 3.29 [2.19, 5.48] | 2.97 [2.06, 4.49] | 4.35 [2.58, 8.17] | <0.001 |
| Monocyte, 10^9^/L (median [IQR]) | 0.43 [0.32, 0.60] | 0.40 [0.30, 0.54] | 0.52 [0.36, 0.73] | <0.001 |
| White blood cell, 10^9^/L (median [IQR]) | 6.41 [5.04, 8.13] | 6.44 [5.15, 7.99] | 6.35 [4.76, 8.48] | 0.044 |
| Platelet, 10^9^/L (median [IQR]) | 198.00 [147.00, 253.00] | 201.00 [153.00, 253.00] | 191.00 [136.00, 253.00] | <0.001 |
| Hemoglobin, g/L (mean (SD)) | 106.42 (30.07) | 111.01 (29.21) | 97.92 (29.81) | <0.001 |
| LDH, U/L (median [IQR]) | 216.00 [177.30, 270.00] | 203.00 [170.00, 244.40] | 245.00 [198.00, 323.00] | <0.001 |
| BUN, mmol/L (median [IQR]) | 12.17 [6.54, 21.00] | 11.13 [6.27, 19.91] | 13.97 [7.25, 22.94] | <0.001 |
| Uric acid, umol/L (mean (SD)) | 412.09 (141.53) | 408.18 (136.55) | 419.32 (150.08) | 0.001 |
| Creatinine, umol/L (median [IQR]) | 325.00 [111.00, 709.00] | 379.00 [123.00, 787.00] | 254.00 [96.00, 597.75] | <0.001 |
| eGFR, mL/min/1.73m^2^ (median [IQR]) | 23.60 [7.60, 69.20] | 26.00 [8.10, 71.25] | 18.60 [7.00, 64.30] | <0.001 |
| CKD stage (%) |  |  |  | <0.001 |
| 1 | 1189 (15.4) | 814 (16.2) | 375 (13.9) |  |
| 2 | 1072 (13.9) | 715 (14.3) | 357 (13.2) |  |
| 3 | 1251 (16.2) | 833 (16.6) | 418 (15.4) |  |
| 4 | 954 (12.4) | 634 (12.7) | 320 (11.8) |  |
| 5 | 3250 (42.1) | 2014 (40.2) | 1236 (45.7) |  |
| Total bilirubin, umol/L (median [IQR]) | 5.00 [3.50, 7.30] | 4.96 [3.50, 7.10] | 5.10 [3.60, 7.60] | <0.001 |
| Globulin, g/L (median [IQR]) | 31.70 [27.00, 38.15] | 32.90 [27.90, 39.10] | 29.70 [25.20, 35.70] | <0.001 |
| Albumin, g/L (mean (SD)) | 37.80 (14.42) | 40.19 (14.82) | 33.36 (12.48) | <0.001 |
| Calcium iron, mmol/L (mean (SD)) | 2.12 (0.24) | 2.17 (0.23) | 2.03 (0.23) | <0.001 |
| Chlorine iron, mmol/L (mean (SD)) | 101.70 (5.49) | 103.01 (4.82) | 99.29 (5.84) | <0.001 |
| Sodium iron, mmol/L (median [IQR]) | 139.00 [136.20, 141.00] | 139.70 [137.50, 141.50] | 136.90 [132.62, 139.60] | <0.001 |
| Potassium iron, mmol/L (mean (SD)) | 4.48 (0.76) | 4.41 (0.73) | 4.61 (0.81) | <0.001 |
| ACR, ug/mg (median [IQR]) | 1310.10 [333.00, 3169.00] | 1034.20 [278.00, 2806.30] | 1758.20 [470.82, 3852.02] | <0.001 |
| D-dimer, mg/L (median [IQR]) | 0.84 [0.42, 1.85] | 0.67 [0.38, 1.40] | 1.34 [0.61, 2.84] | <0.001 |
| INR (median [IQR]) | 1.02 [0.96, 1.10] | 1.01 [0.96, 1.08] | 1.04 [0.97, 1.13] | <0.001 |
| PT, s (median [IQR]) | 13.30 [12.70, 14.00] | 13.20 [12.70, 13.90] | 13.50 [12.80, 14.50] | <0.001 |
| PTA, % (mean (SD)) | 97.24 (18.39) | 99.28 (16.30) | 93.45 (21.23) | <0.001 |
| TT, s (median [IQR]) | 17.10 [16.30, 18.30] | 17.10 [16.30, 18.20] | 17.20 [16.30, 18.50] | 0.458 |
| APTT, s (median [IQR]) | 37.90 [34.90, 41.60] | 37.50 [34.70, 40.60] | 39.10 [35.10, 44.20] | <0.001 |
| Fibrinogen, g/L (mean (SD)) | 4.59 (1.64) | 4.36 (1.48) | 5.01 (1.82) | <0.001 |
| Urine ph (mean (SD)) | 6.34 (0.78) | 6.25 (0.75) | 6.51 (0.82) | <0.001 |
| Urine WBC, /ul (median [IQR]) | 11.10 [5.00, 32.00] | 10.20 [4.50, 26.00] | 15.40 [6.60, 44.50] | <0.001 |
| Urine RBC, /ul (median [IQR]) | 24.50 [8.90, 84.80] | 21.00 [7.80, 67.70] | 32.20 [11.33, 116.40] | <0.001 |
| Clinical outcomes |  |  |  |  |
| LOS, d (median [IQR]) | 9.00 [6.00, 13.00] | 8.00 [6.00, 11.00] | 10.00 [7.00, 15.00] | <0.001 |
| Total costs, yuan (median [IQR]) | 11786.71 [7958.41, 20696.87] | 10448.17 [7243.54, 15573.89] | 17049.90 [10041.23, 33008.09] | <0.001 |
| PE (%) | 819 (10.6) | 23 (0.5) | 796 (29.4) | <0.001 |
| Death within 14 days (%) | 233 (3.0) | 9 (0.2) | 224 (8.3) | <0.001 |
| Death within 30 days (%) | 375 (4.9) | 20 (0.4) | 355 (13.1) | <0.001 |
| Hospital mortality (%) | 414 (5.4) | 23 (0.5) | 391 (14.4) | <0.001 |

Data were presented by numbers (%) or median (IQR) or mean (SD).

ANCA, anti-neutrophil cytoplasmic antibodies; CNI, calcineurin inhibitor; NLR, neutrophil to lymphocyte ratio; LDH, lactate dehydrogenase; BUN, blood urea nitrogen; eGFR, estimated glomerular filtration rate; ACR, urine albumin creatinine ratio; INR, international normalized ratio; PT, prothrombin time; PTA, prothrombin time activity; TT, thrombin time; APTT, activated partial thromboplastin time; WBC, white blood cell; RBC, red blood cell; LOS, length of stay; PE, primary endpoint; IQR, interquartile range; SD, standard Deviation.

‡Chronic nephritis syndrome represented patients without pathological diagnosis.

*Anti-proliferative agents include mycophenolate mofetil, leflunomide, cyclophosphamide, methotrexate, azathioprine.

†Calcineurin inhibitor include cyclosporine, tacrolimus.

Supplementary Table 2 Multivariate logistic regression of risk factors for pneumonia in CKD patients.

| Variables | OR (95%CI) | P value |
| --- | --- | --- |
| ANCA-associated nephritis | 2.16 (1.02-4.58) | 0.04 |
| Fever | 16.35 (11.37-23.72) | <0.001 |
| Cough | 12.62 (8.07-19.95) | <0.001 |
| Expectoration | 14.68 (8.00-28.08) | <0.001 |
| Dyspnea | 7.97 (5.24-12.20) | <0.001 |
| Chest pain | 9.03 (4.22-19.59) | <0.001 |
| Ground glass opacity | 17.11 (12.86-22.92) | <0.001 |
| Reticular shadow | 8.42 (5.21-13.76) | <0.001 |
| Pleural effusion | 2.09 (1.59-2.75) | <0.001 |
| Glucocorticoid | 1.60 (1.25-2.03) | <0.001 |
| Biological agents | 2.43 (1.17-5.00) | 0.02 |
| Heart rate, beat/min | 1.01 (1.00-1.02) | 0.03 |
| Diastolic blood pressure, mmHg | 1.01 (1.00-1.02) | 0.02 |
| Neutrophil, 10^9^/L | 1.26 (1.11-1.44) | <0.001 |
| Lymphocyte, 10^9^/L | 1.29 (1.01-1.66) | 0.04 |
| NLR | 1.06 (1.03-1.09) | <0.001 |
| Monocyte, 10^9^/L | 11.06 (6.62-18.61) | <0.001 |
| White blood cell, 10^9^/L | 0.67 (0.59-0.76) | <0.001 |
| Hemoglobin, g/L | 0.98 (0.96-0.99) | <0.001 |
| Creatinine, umol/L | 1.00 (1.00-1.00) | <0.001 |
| Globulin, g/L | 0.96 (0.95-0.97) | <0.001 |
| Albumin, g/L | 0.98 (0.97-0.99) | <0.001 |
| Calcium iron, mmol/L | 0.41 (0.24-0.70) | <0.001 |
| Chlorine iron, mmol/L | 0.91 (0.89-0.93) | <0.001 |
| Sodium iron, mmol/L | 0.97 (0.97-0.98) | <0.001 |
| Potassium iron, mmol/L | 1.38 (1.18-1.61) | <0.001 |
| ACR, ug/mg | 1.00 (1.00-1.00) | 0.01 |
| Urine ph | 1.41 (1.23-1.62) | <0.001 |

ANCA, anti-neutrophil cytoplasmic antibodies; NLR, neutrophil to lymphocyte ratio; ACR, urine albumin creatinine rati

Supplementary Figure 1 Performance of the severity of the PNPI score for predicting PE.


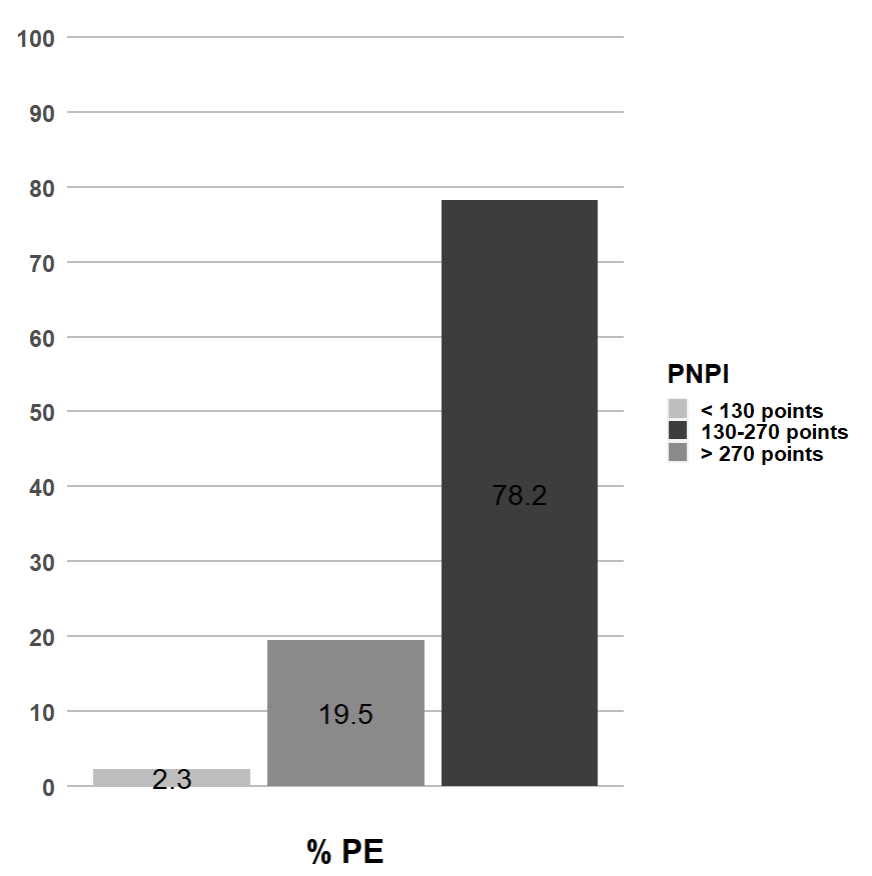


Supplementary Figure 2 Receiver operating characteristics for severity scores at enrollment regarding PE prediction in training set.


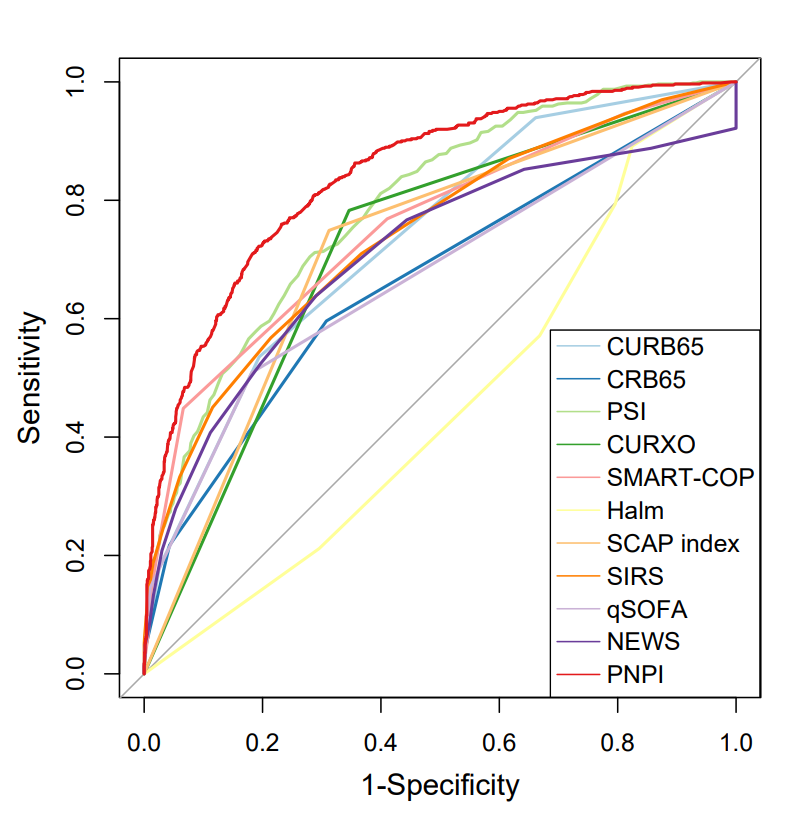


Supplementary Figure 3 Calibration curve and DCA of nomogram in training set. DCA: decision curve analysis A: Calibration curve; B: DCA


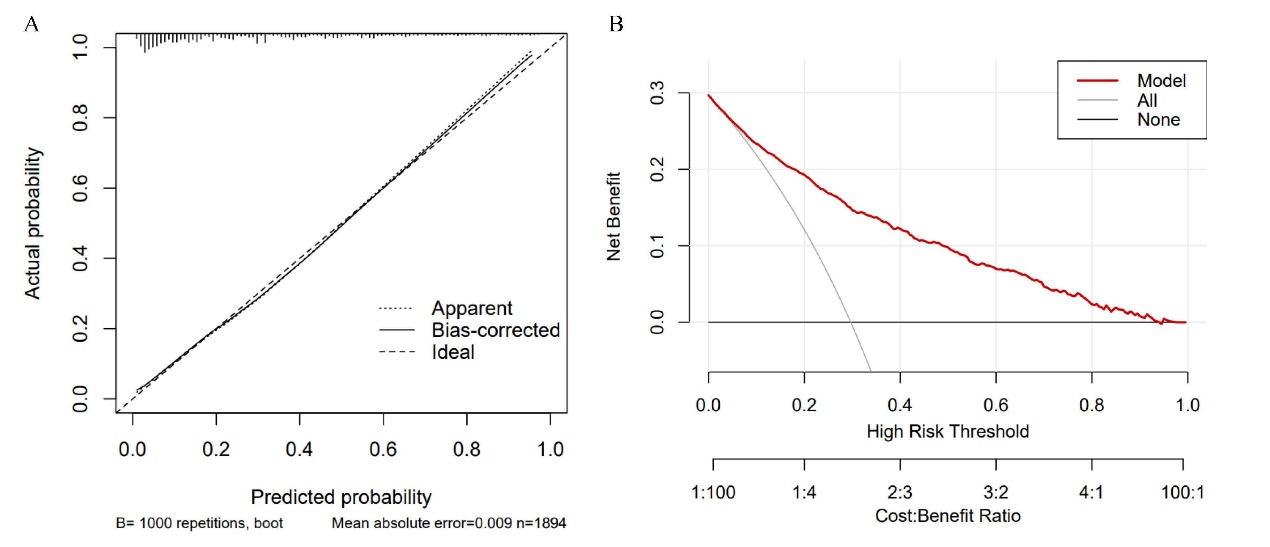

Supplement: Supplementary file 1 [file Data_Sheet_1.docx]
